# Supplementary material for: The Interactions of H2TMPyP, Analogues and Its Metal Complexes with DNA G-Quadruplexes—An Overview
Source: Biomolecules. 2021 Sep 25;11(10):1404. doi: 10.3390/biom11101404 (PMC8533071; doi:10.3390/biom11101404)
Supplement: Supplementary file 1 [file biomolecules-11-01404-s001.zip › biomolecules-1378033-supplementary.pdf]

The interactions of H_2TMPyP, analogues and its metal complexes with G-quadruplex DNA – an overview

Catarina I. V. Ramos[1,*], Ana R. Monteiro[1,2], Nuno M. M. Moura[1], M. Amparo F. Faustino[1], Tito Trindade[2] and M. Graça P. M. S. Neves[1]

1 LAQV-REQUIMTE, Department of Chemistry, University of Aveiro, 3810-193 Aveiro Portugal;
2 CICECO-Aveiro, Institute of Materials, Department of Chemistry, University of Aveiro, 3810-193 Aveiro, Portugal

* Correspondence: c.ramos@ua.pt; Tel.: +351 234 370 692

Received: date; Accepted: date; Published: date

Supporting Material

1. Material and Methods

1.1. Chemicals

The chemicals were purchased as analytical grade and used without further purification and, when required, the solutions were prepared in MilliQ water. 5,10,15,20-Tetrakis(1-methylpyridinium-4-yl)porphyrin tosylate (H_2TMPyP) was purchased from Sigma Aldrich (Aldrich, Steinheim, Germany), and the lyophilized DNA oligonucleotides were purchased from ThermoFisher Scientific (Waltham, MA, USA). The metalloporphyrins MTMPyP (M = Ag^{II}, Zn^{II}, Co^{III}, Ni^{II}, Pd^{II}, Mn^{III} and Cu^{II}) were synthesized and characterized as previously described [**Error! Bookmark not defined.**]. The molar extinction coefficients of the porphyrins were determined in phosphate-buffered saline (PBS) and are presented in Table S1. For H_2TMPyP and oligonucleotides, the provided molar extinction coefficient values were considered. Stock solutions of the porphyrins were prepared in DMSO and stored at 4 °C. Before each assay, an aliquot of the stock solution was diluted to a final concentration of 2.0 μM in PBS buffer.

1.2. Preparation of DNA structures (double chain and G-quadruplexes)

A PBS solution, containing 20 mM of phosphate buffer (10 mL of KH_2PO_4 1 M, and 200 μL of K_2HPO_4 1 M), 0.1 mM of ethylenediaminetetraacetic acid (EDTA), and 100 mM of KOH was prepared with pH adjusted to 6.8. The PBS solution was used as the solvent for oligonucleotide solutions. After solubilization in PBS, each oligonucleotide was heated up to 85 °C for 10 min and left to cool *overnight* to assure the correct folding into double chain or GQ structures. The solutions were stored at 20 °C.

1.3. Synthesis of the Ag^{II}TMPyP complex

The Ag(II) complex of TMPyP was prepared by modification of a procedure previously described in the literature [1]. TMPyP (15 mg, 2.4 x 10^{-5} mol) was dissolved in DMF and $AgNO_3$ (4.1 mg, 2.4 x 10^{-5} mol) was added to the solution and the mixture was stirred at 80 °C for 24 h. After, the metalloporphyrin was precipitated with diethyl ether and the resulting solid was washed with a diethyl ether/propan-1-ol (2:1) mixture. The desired complex was dried in the oven at 40 °C for 24 h. The structure of the Ag^{II}TMPyP was confirmed by mass

spectrometry; the mass spectrum displays a *m/z* peak at 196.3 corresponding to the $[M]^{4+}$ ion (Figure S5, SI).

1.4. UV-Vis spectroscopy

UV-Vis absorption spectra were recorded in a UV-2501-PC spectrophotometer (Shimadzu Corporation, Kyoto, Japan) in the range between 350–700 nm, using a reduced quartz cuvette of 1 cm length at controlled temperature (25 °C) by using a Compatible Control CC1 (Huber, Huntersville, NC, USA). During the spectroscopic titrations, all the compounds were dissolved in PBS to mimic the physiological conditions. These titrations were performed by successive additions of the oligonucleotide solution to each porphyrin solution (1 mL) with an initial concentration of 2 μM, and the respective spectrum was recorded in the range of 350–700 nm. The spectra were mathematically corrected for the dilution effect and to ensure the reproducibility of results, all the experiments were performed in triplicate. According to the literature, titrations were finished after 3–5 values of constant absorbance [2].

The binding constants (Kb) were determined by fitting data obtained from the UV-Vis absorption titrations to the equation (1)

$$\frac{[DNA]}{\varepsilon a-\varepsilon b} = \frac{[DNA]}{\varepsilon a-\varepsilon b} + \frac{1}{Kb(\varepsilon a-\varepsilon b)} \qquad (1)$$

where [DNA] is the concentration of DNA, and εa, εf and εb correspond to apparent extinction coefficients for the complex in the presence, in the absence and to fully bound DNA, respectively [ ]. The Kb was then calculated from the ratio of the slope to the intercept.

The percentage of hypochromicity of the absorption band was calculated using the following equation %hypochromicity = $[(\varepsilon_{free} - \varepsilon_{bound})/\varepsilon_{free}]$ x100, where ε_{bound} was calculated using the Beer's Law ($\varepsilon_{bound} = A_{bound}/C_{bound}$) and ε_{free} are the molar extinction coefficient values reported in the literature for H_2TMPyP $\varepsilon_{420} = 226000\ M^{-1}cm^{-1}$ and experimentally obtained, in PBS, for the metalloporphyrins.

Method of continuous variation, or Job plot method, [3] was used to obtain the binding stoichiometries according to expression (2). Job diagrams were obtained by plotting F(χ) against the molar fraction of porphyrin (χ), according to the expression:

$$F(\chi) = \frac{A_{obs}-(C_{lig}.\varepsilon_{lig}+C_{DNA}.\varepsilon_{DNA})}{C_{lig}+C_{DNA}} \qquad (2)$$

where A_{obs} corresponds to the observed absorbance of the solution after each DNA addition, C_{lig} and C_{DNA} correspond, respectively, to the total concentration of porphyrin and DNA, and ε_{lig} and ε_{DNA} are the extinction coefficient of the porphyrin and DNA structure, respectively, at a given wavelength. The stoichiometry corresponds to the maximum or minimum observed.

2.5. Fluorescence spectroscopy

Stock solutions of 35 μM of TO and 10 μM of the different oligonucleotides were prepared. The previous solutions were mixed to obtain the desired TO-oligonucleotide solution and after 10 min of orbital shaking at 500 rpm, the fluorescence was measured in a Fluoromax-3 spectrofluorometer (Horiba), using excitation wavelength at 485 nm and emission range of 510-750 nm. Excitation and emission slits were set at 10 nm. The obtained fluorescence data were considered as FA0. Solutions with increasing concentrations (0 to 4.0 μM) of each ligand were prepared and added to the TO-oligonucleotide ones and the fluorescence was measured using the same experimental parameters. The percentage of displacement, DC_{50} was calculated using the equation:

$$DC_{50} = 100 - \frac{F_A}{F_{A0}} \times 100$$

where $F_A = F - F_{H2O}$ and $F_{A0} = F_0 - F_{H2O}$, where F is the fluorescence intensity of each sample, F_{H2O} is the fluorescence intensity of milli-Q, and F_0 the fluorescence from the fluorescent probe bound to DNA without added ligand.

2. Results

Table S1. Structures of the studied porphyrins, relevant photophysical parameters.

	Ligand	M	Ar	ε ($M^{-1}cm^{-1}$)	λ_{max} (nm)
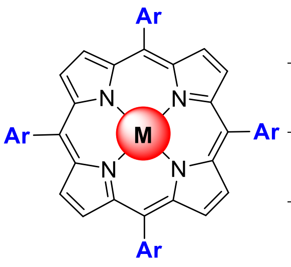	H_2TMPyP	H_2	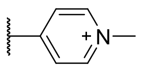	226000	421
	Zn^{II}TMPyP	Zn		225000	436
	Co^{III}TMPyP	Co		83429	434
	Ni^{II}TMPyP	Ni		99161	416
	Pd^{II}TMPyP	Pd		196970	416
	Ag^{II}TMPyP	Ag		136014	430
	Mn^{III}TMPyP	Mn		144875	461
	Cu^{II}TMPyP	Cu		456283	420

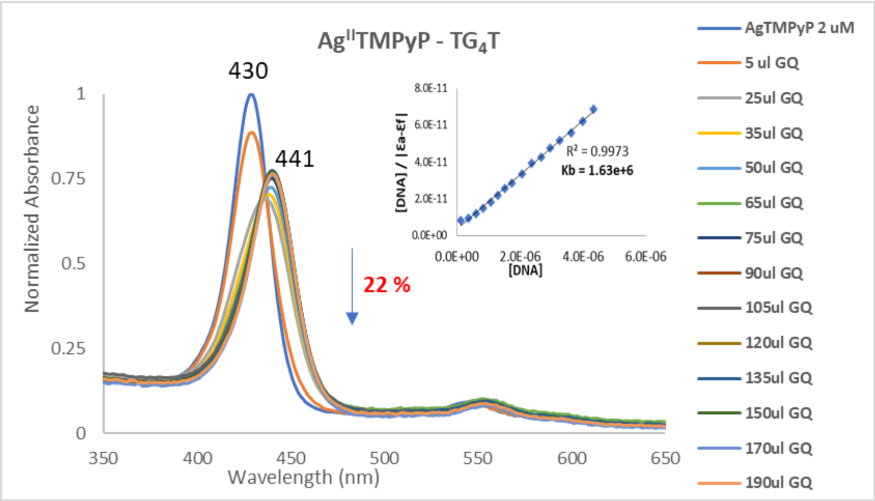

Figure S1. UV-Vis spectra obtained from titration of **Ag^{II}TMPyP** with the tetramolecular GQ DNA structure TG$_4$T. Inset - binding constant (Kb).

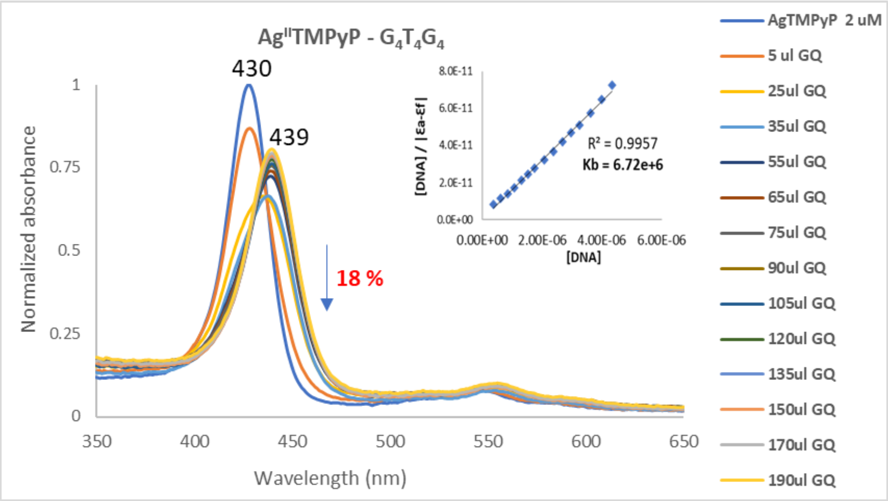

Figure S2. UV-Vis spectra obtained from titration of **Ag^{II}TMPyP** with the bimolecular GQ DNA structure G$_4$T$_4$G$_4$. Inset represents Job Plot applied to obtain the binding constants (Kb).

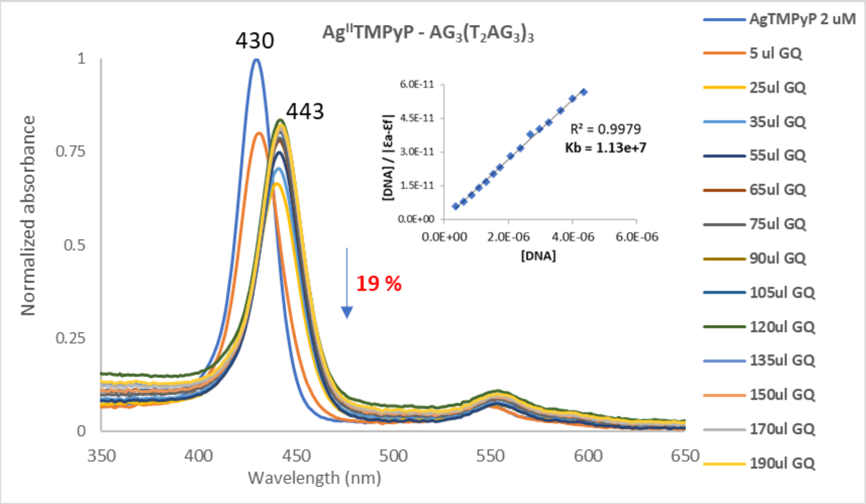

Figure S3. UV-Vis spectra obtained from titration of **Ag^{II}TMPyP** with the unimolecular GQ DNA structure $AG_3(T_2AG_3)_3$. Inset represents Job Plot applied to obtain the binding constants (Kb).

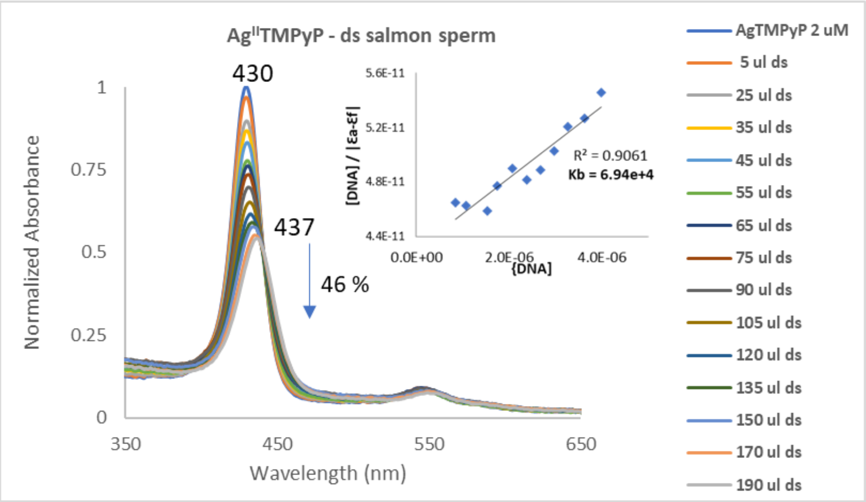

Figure S4. UV-Vis spectra obtained from titration of **Ag^{II}TMPyP** with the double-stranded DNA structures from salmon sperm. Inset represents Job Plot applied to obtain the binding constants (Kb).

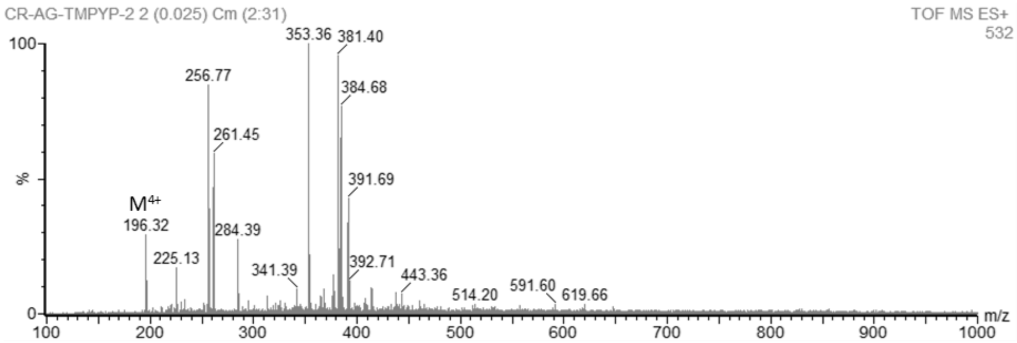

Figure S5. Mass spectrum of the **Ag^{II}TMPyP** derivative.

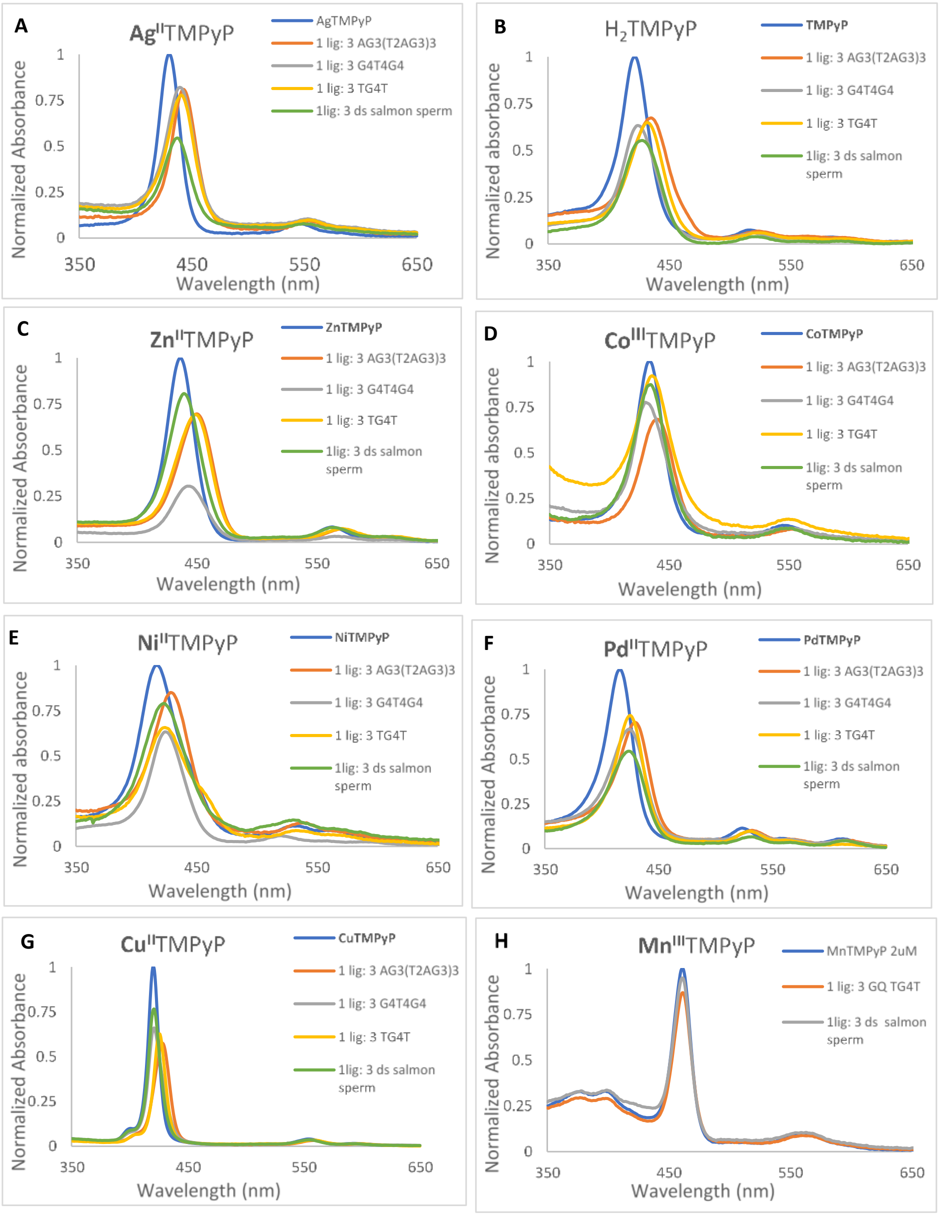

Figure S6. Comparative UV-Vis absorption spectra (350–650 nm) at the end of titration (1:3 L:DNA) of the solution of A) Ag^{II}TMPyP, B) H_2TMPyP, C) Zn^{II}TMPyP, D) Co^{III}TMPyP E) Ni^{II}TMPyP, F) Pd^{II}TMPyP, G) Cu^{II}TMPyP and H) Mn^{II}TMPyP (2.0 μM) with the selected unimolecular, bimolecular and tetramolecular GQ structures and salmon sperm double-stranded DNA. DNA structures were prepared in 20 mM PBS buffer with 100 mM KCl.

References:

[1] Tovmasyan A.; Babayan N.; Poghosyan D.; *et al.* Novel amphiphilic cationic porphyrin and its Ag(II) complex as potential anticancer agents. *J. Inorg. Biochem.* **2014**, *140*, 94 https://pubmed.ncbi.nlm.nih.gov/25086237/

[2] Nagesh N.; Sharma V.K.; Ganesh K.A.; Lewis E.A. Effect of ionic strength on porphyrin drugs interaction with quadruplex DNA formed by the promoter region of C-myc and Bcl2 oncogenes. *J. Nucleic Acids* **2010**, 2010 https://doi.org/10.4061/2010/146418

[3] Hill Z. D.; MacCarthy P. Novel approach to Job's method: An undergraduate experiment. *J. Chem. Educ.* **1986**, *63*, 162 https://pubs.acs.org/doi/abs/10.1021/ed063p162
